# The incidence, risk factors, and long-term outcomes of acute kidney injury in hospitalized diabetic ketoacidosis patients

**DOI:** 10.1186/s12882-020-1709-z

**Published:** 2020-02-12

**Authors:** Junzhe Chen, Honghui Zeng, Xia Ouyang, Mingsheng Zhu, Qiuyan Huang, Wenjuan Yu, Li Ling, Hui-yao Lan, Anping Xu, Ying Tang

**Affiliations:** 1 Department of Nephrology, Sun Yat-sen Memorial Hospital, Sun Yat-sen University, 107 Yanjiang West Road, Guangzhou, China; 2 Guangdong Provincial Key Laboratory of Malignant Tumor Epigenetics and Gene Regulation, Sun Yat-sen Memorial Hospital, Sun Yat-sen University, Guangzhou, China; 3 Department of Nephrology, The people’s Hospital of Gaozhou, Gaozhou, Guangdong China; 4 Faculty of Medical Statistics and Epidemiology, School of Public Health, Sun Yat-sen University, Guangzhou, China; 5 Department of Medicine and Therapeutics, Li KaShing Institute of Health Sciences, The Chinese University of Hong Kong, Hong Kong, China

**Keywords:** Diabetic ketoacidosis, Acute kidney injury, Chronic kidney disease, Mortality

## Abstract

**Background:**

Emerging evidence has demonstrated that acute kidney injury (AKI) is an important risk factor associated with increased morbidity and mortality in diabetic ketoacidosis (DKA) patients. The current study aimed to investigate the incidence rate, risk factors, long-term renal outcomes, and mortality in DKA patients with AKI.

**Methods:**

A total of 179 patients diagnosed with DKA at Sun Yat-sen Memorial Hospital from January 2012 to January 2018 were included in the analysis. AKI was diagnosed according to the 2012 KDIGO criteria. Risk factors, long-term renal outcomes, and mortality were analyzed by logistic regression and Cox proportional hazards models.

**Results:**

Among 179 DKA patients, 98 patients (54.75%) were diagnosed as AKI. Aging; increased blood glucose, serum uric acid and white blood cells; decreased serum pH and albumin; coma; and preexisting chronic kidney disease (CKD) were risk factors of AKI in patients with DKA. During follow-up, DKA patients with AKI showed more than a two-fold decline in eGFR within 1 year after discharge from the hospital when compared with non-AKI DKA patients. Furthermore, AKI was also an independent risk factor for poor long-term renal outcomes and mortality in DKA patients.

**Conclusions:**

Multiple risk factors contribute to the development of AKI in DKA patients. AKI and advanced AKI stage are associated with rapid progressive CKD and long-term mortality in patients with DKA.

## Background

Diabetic ketoacidosis (DKA), a severe complication of diabetes mellitus (DM), is the leading cause of hospitalization, morbidity and mortality in patients with DM [1, 2]. DKA is associated with hyperglycemic crises and featured by metabolic acidosis, the production of ketoacids, volume depletion, and electrolyte imbalance. Due to glucose-induced osmotic polyuria and even emesis, volume depletion is a major cause of acute kidney injury (AKI) in DKA patients [3]. It is now well accepted that AKI is an important factor than influences long-term morbidity and mortality [4, 5]. The clinical manifestations of AKI range from a mild increase in serum creatine (SCr) to anuric renal failure requiring dialysis. Most of the available studies focused on patients with acute renal failure (ARF) caused by DKA, and those with mild renal impairment who did not meet the criteria of ARF were overlooked. Brenden E et al. found that 44 of 106 (41.5%) DKA combined with AKI patients did not have documentation of AKI resolution prior to discharge [1]. To date, no study has investigated the effect of AKI on long-term renal outcomes in DKA patients after discharge. To our knowledge, this is the first study to investigate the association between AKI and long-term renal outcomes in DKA patients. The objective of this study was to determine the effects of AKI on long-term outcomes, including renal function and mortality, in DKA patients and to explore the possible risk factors associated with AKI in DKA patients.

## Methods

### Study design and participants

All hospitalized patients aged more than 18 years who were diagnosed with DKA at Sun Yat-sen Memorial Hospital of Sun Yat-sen University from January 2012 to January 2018 were retrospectively reviewed. Hyperglycemia patients with blood glucose> 11 mmol/L, acidosis with a serum pH < 7.3 or bicarbonate level < 15 mmol/L and elevation of serum or urine ketones were diagnosed with DKA [1]. Patients with CKD (stage 5), end-stage renal disease, dialysis or incomplete medical records were excluded. The hospital ethics committee approved this study (SYSEC-KY-KS-2019-135) and waived the need for patients’ written informed consent. We obtained oral informed consent from patients when we conducted a visit by telephone which was approved by our ethics committee. The study was conducted in accordance with the Declaration of Helsinki (2013).

### Procedures

We reviewed the hospital electronic records and collected the anonymized baseline characteristics of DKA patients, including demographic characteristic, disease history, physical examination, and biological examination data. All patients were followed by either clinical medical data reviews or enquiry via telephone.

### Measurements

#### Baseline characteristics

Electronic records were reviewed, and detailed information of DKA patients including sex, age, height, weight, body mass index (BMI), type of DM, history of cardiovascular disease (CVD) and preexisting CKD, was recorded. Physical examination data, including blood pressure (systolic blood pressure-SBP and diastolic blood pressure-DBP), temperature, heart rate and mental status on admission, were recorded. Coma refers to the clinical state in which a patient is unarousable and does not respond to stimuli [6]; it is a life-threatening diabetes complication and can be caused by DKA. Biological parameters, including pH, SCr, blood glucose (Glu), serum ketone, serum albumin (Alb), serum uric acid (SUA), white blood cells (WBCs), and glycosylated hemoglobin (HbA1c), were collected on admission. At the same time, all SCr data while in the hospital were collected. The estimated glomerular filtration rate (eGFR) was calculated using the CKD-EPI formula [7]. The admission and discharge eGFRs were calculated using admission and discharge SCr levels. Glu, serum ketones, Alb, SUA, and SCr in blood samples were measured by an automated biochemical analyzer 5800 (BECKMAN) or 7600 (HITACHI). WBCs were measured by a blood routine analyzer (SYSMEX XN-2100), and pH was tested in arterial blood by a blood gas analyzer (PHOX U). The qualitative examination of urinary protein was accomplished by a SYSMEX AUTION MAX-4030. HbA1c was examined by glycosylated hemoglobin analyzer (BIO-RAD VARIANT II).

#### Outcome measures

The diagnosis of AKI was defined as an increase of SCr ≥ 26.5 μmol/L within 48 h, or a 1.5-fold increase in SCr above the baseline value within 7 days [8, 9]. The severity of AKI was classified into three grades by referring to the peak SCr relative to the baseline SCr according to the Kidney Disease Improving Global Outcomes (KDIGO) criteria [8, 9]. In our study, we chose the minimum value of SCr (SCrmin) in the hospital as the baseline level for the follow-up analysis. SCr and mortality were recorded during follow-up. The eGFR decline rate in each patient was calculated as following: $\frac{\mathrm{Follow}-\mathrm{up}\ \mathrm{eGFR}\left(\mathrm{ml}/\min /1.73\mathrm{m}2\right)-\mathrm{Baseline}\ \mathrm{eGFR}\left(\mathrm{ml}/\min /1.73\mathrm{m}2\right)}{\mathrm{Baseline}\ \mathrm{eGFR}\left(\mathrm{ml}/\min /1.73\mathrm{m}2\right)\times \mathrm{Time}\left(\mathrm{year}\right)}$. Diabetes patients with an eGFR decline over 4.0% per year were defined as the rapid decliner group; rapid decliners were associated with more severe renal dysfunction and higher mortality than nonrapid decliners during a 10-year follow-up period [10]. Therefore, we chose this index to represent the long-term renal outcome, and all patients were divided into two groups: the rapid decliner group or the nonrapid decliner group.

### Statistical analysis

The baseline characteristics of the DKA patients were summarized by descriptive statistics. Continuous variables with symmetric distribution were expressed as the mean ± standard deviation (SD), and as the median (interquartile range) for those with asymmetric distribution. Categorical variables were reported as proportions of the number of patients. Student’s t-test, Wilcoxon rank sum test, chi-square test or correction for continuity were used to compare data between the AKI and non-AKI groups. Student’s t-test was used for continuous normally distributed parameters, and Wilcoxon rank sum test was used for continuous nonnormally distributed parameters. Statistical significance of differences between categorical variables was valuated using the chi-square test. At the same time, when one of the theoretical frequencies was less than 5, correction for continuity was used. Logistic regression was used to evaluate risk factors associated with AKI. Cox proportional hazards model was used to identify risk factors associated with long-term renal outcomes and mortality. Risk factors found to be statistically significant in the univariate analyses (*P* < 0.1) were then tested in the multivariate analysis using logistic regression (Forward, Logistic regression-LR) or Cox proportional hazards (Forward, LR) modeling. Only the parameters that showed a significant *p*-value in the multivariate analysis are presented. Kaplan-Meier analysis and the log-rank test were used to compare long-term renal outcomes and mortality between different AKI stages according to the KDIGO criteria. *P* < 0.05 from two-sided tests was considered statistically significant. Analysis was performed using IBM-SPSS version 19 (IBM Corporation, Armonk, New York, USA).

## Results

A total of 209 hospitalized patients diagnosed with DKA at Sun Yat-sen Memorial Hospital of Sun Yat-sen University from January 2012 to January 2018 were systematically retrospectively reviewed. A total of 179 DKA patients (85.6%), with an average age of 40 years, were included in our study. Thirty DKA patients without at least two blood biochemistry results, which were required for AKI diagnosis in the hospital, were excluded. A total of 95 (53.1%) patients were male. Considering the 2012 KDIGO guidelines [8], 98 patients (54.8%) developed AKI; 66 (67%) presented with stage 1, 22 (22%) presented with stage 2, and 10 (10%) presented with stage 3. Unexpectedly, more than 90% of AKI patients were underdiagnosed in the hospital according to the discharge diagnosis, determined via medical records retrievals.

### The baseline characteristics of the AKI and non-AKI groups

Table 1 showed that the AKI group was older and had a higher incidence of CVD and CKD than the non-AKI group (*P* < 0.05). Increased heart rate, incidence of coma on admission and proteinuria were recorded in the AKI group compared to the non-AKI group (*P* < 0.05). Our results showed that AKI patients had higher blood glucose, SUA and WBCs and lower pH and Alb levels than non-AKI patients (*P* < 0.05).

**Table 1:** The baseline characteristics of DKA patients between non-AKI and AKI group

Parameters	Total (*n* = 179)	AKI group (*n* = 98)	Non-AKI group (*n* = 81)	P
Male, n (%)	95 (53.07)	58 (59.18)	37 (45.67)	0.072
Age (year)	40.30 ± 19.47	53.46 ± 20.13	44.27 ± 17.46	0.002
Body Mass Index (kg/m^2^)	22.29 ± 4.19	22.40 ± 3.73	22.16 ± 4.70	0.709
History of CVD, n(%)	27 (15.08)	20 (20.41)	7 (8.64)	0.029
Type 2 diabetic mellitus, n(%)	110 (61.45)	66 (67.35)	44 (54.32)	0.075
Preexisting CKD, n(%)	20 (11.17)	16 (16.33)	4 (4.94)	0.030
General condition of admission				
Systolic blood pressure-SBP (mmHg)	126.26 ± 24.30	127.56 ± 26.61	124.68 ± 21.23	0.431
Diastolic blood pressure-DBP (mmHg)	75.26 ± 13.45	74.02 ± 13.88	76.77 ± 12.82	0.175
Temperature(°C)	36.80 [36.40–37.20]	36.90 [36.50–37.43]	36.70 [36.30–37.10]	0.100
Heart rate (times/min)	99.80 ± 20.63	103.66 ± 22.12	95.13 ± 17.71	0.005
Coma, n(%)	22 (12.29)	20 (20.41)	2 (2.47)	0.001
Biochemical indicator of admission				
Glu (mmol/L)	28.01 ± 10.47	31.25 ± 11.45	24.09 ± 7.52	< 0.001
Serum ketone (mmol/L)	2.00 [1.00–4.54]	2.05 [1.00–4.56]	1.80 [1.00–4.51]	0.376
pH	7.32 [7.24–7.37]	7.30 [7.16–7.36]	7.34 [7.29–7.38]	0.002
HbA1c(%)	11.98 ± 2.82	11.96 ± 2.94	12.01 ± 2.70	0.912
SUA (μmol/L)	326.73 ± 148.92	356.46 ± 159.95	290.77 ± 126.23	0.003
Alb(g/L)	31.88 ± 7.14	29.98 ± 6.60	34.18 ± 7.12	< 0.001
WBCs(10^9^/L)	12.61 [8.13–19.76]	16.51 [11.40–21.76]	9.38 [6.12–15.20]	< 0.001
Proteinuria, n(%)	31 (17.32)	22 (22.45)	9 (11.11)	0.046
SCr on admission (μmol/L)	133.16 ± 80.29	170.14 ± 92.28	88.43 ± 17.81	< 0.001
eGFR on admission (ml/min/1.73m^2^)	60.25 ± 27.62	42.37 ± 17.03	81.87 ± 21.92	< 0.001
Biochemical indicator at discharge				
SCr at discharge (μmol/L)	88.61 ± 33.11	97.07 ± 40.66	78.43 ± 15.57	< 0.001
eGFR at discharge (ml/min/1.73m^2^)	84.81 ± 27.06	78.78 ± 29.72	92.11 ± 21.44	0.001

### The risk factors of AKI in DKA patients

The multivariate logistic regression identified that older age [odds ratio-OR (95% confidence interval-CI) 1.033 (1.009–1.058), *P* = 0.008]; increased Glu [OR (95%CI) 1.087 (1.034–1.142), *P* = 0.001], SUA [OR (95%CI) 1.006 (1.002–1.009), *P* = 0.001], and WBC [OR (95%CI) 1.089 (1.026–1.157), *P* = 0.005]; and decreased pH [OR (95%CI) 0.001 (0.000–0.080), *P* = 0.002], and serum Alb [OR (95%CI) 0.937(0.881–0.996), *P* = 0.038]; combined with coma on admission [OR (95%CI) 12.389 (1.823–84.185), *P* = 0.010] and preexisting CKD [OR (95%CI) 6.250 (1.461–26.732), *P* = 0.013] were risk factors of AKI in DKA patients (Table 2).

**Table 2:** Risk factors of AKI in DKA patients

Parameters	Logistic analysis			
	Univariate analysis		Multivariate analysis	
	OR (95%CI)	P	OR (95%CI)	P
Male	1.724 (0.952–3.125)	0.072		
Age (year)	1.026 (1.009–1.042)	0.002	1.033 (1.009–1.058)	0.008
Body Mass Index (kg/m^2^)	1.014 (0.944–1.088)	0.708		
History of CVD	2.711 (1.083–6.786)	0.033		
Type 2 diabetic mellitus	1.734 (0.944–3.185)	0.076		
Preexisting CKD	3.756 (1.203–11.732)	0.023	6.250 (1.461–26.732)	0.013
General condition of admission				
SBP (mmHg)	1.005 (0.993–1.017)	0.430		
DBP (mmHg)	0.985 (0.963–1.007)	0.175		
Temperature(°C)	1.113 (0.844–1.468)	0.449		
Heart rate (beats/min)	1.022 (1.006–1.038)	0.007		
Coma	10.128 (2.290–44.798)	0.002	12.389 (1.823–84.185)	0.010
Biochemical indicator of admission				
Glu (mmol/L)	1.084 (1.045–1.125)	< 0.001	1.087 (1.034–1.142)	0.001
Ketone (mmol/L)	1.085 (0.986–1.194)	0.095		
pH	0.014 (0.001–0.178)	0.001	0.001 (0.000–0.080)	0.002
HbA1c(%)	0.994 (0.895–1.104)	0.912		
SUA (μmol/L)	1.003 (1.001–1.006)	0.004	1.006 (1.002–1.009)	0.001
Alb(g/L)	0.913 (0.871–0.958)	< 0.001	0.937 (0.881–0.996)	0.038
WBCs(10^9^/L)	1.107 (1.056–1.160)	< 0.001	1.089 (1.026–1.157)	0.005
Proteinuria	2.316 (1.000–5.363)	0.050		

### The long-term renal outcomes in DKA patients after discharge during the follow-up period

During the average follow-up time of 22 months, 151 patients were visited 203 times due to renal outcome-related causes or mortality. Compared with the baseline SCr and eGFR prior to discharge, the increase in the SCr level and decrease in the eGFR in the AKI group were significantly greater than those in the non-AKI group during follow-up (*P* < 0.05) (Additional file 1: Table S1). The AKI patients were more likely to develop progressive CKD than the non-AKI patients. Our results showed that the average eGFR decline in the DKA patients in the AKI group was − 6.4 ± 5.0 ml/min/1.73m^2^ per year, while that in the DKA patients in the non-AKI group was − 1.9 ± 3.8 ml/min/1.73m^2^ per year during the average follow-up period of 22 months (*P* < 0.01). Notably, the AKI group showed a significant decrease of 10.5 ml/min/1.73m^2^ in the eGFR in the first 6 months to 1 year, whereas the decrease in the eGFR in the non-AKI group was 4.11 ml/min/1.73m^2^ (*P =* 0.001). The deterioration of renal function slowed after 1 year but remained significantly different between the AKI and non-AKI groups.

### The risk factors for long-term renal outcomes in DKA patients

**Fig. 1:**
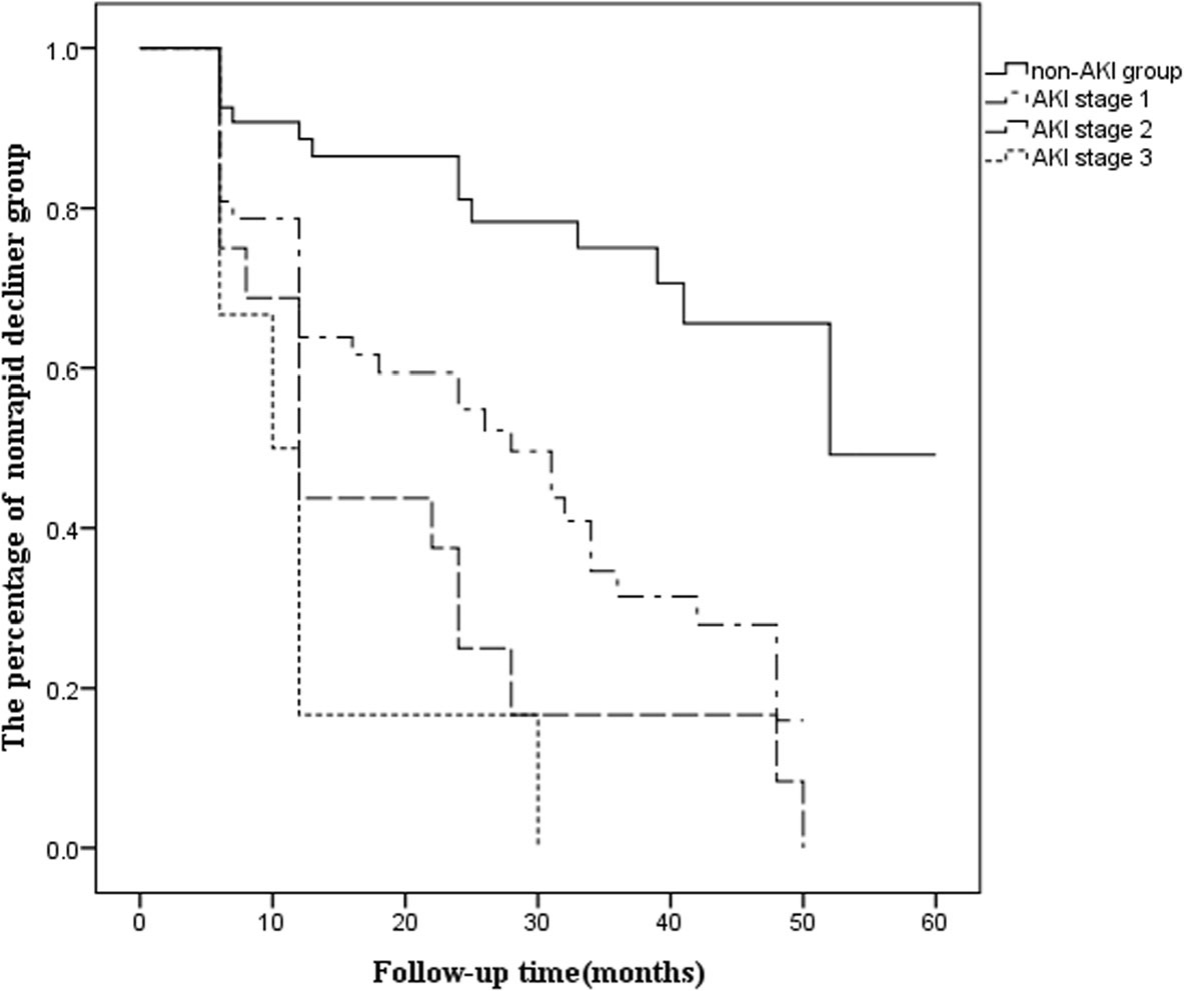
The long-term renal outcomes in DKA patients according to non-AKI and AKI stages. (Log-rank: *P* < 0.001)

The followed patients were divided into two groups based on the decline in the eGFR decline rate. After discharge, DKA patients with an eGFR decline rate of more than 4.0% per year were defined as the rapid decliner group [10] and those with an eGFR decline rate of less than 4% per year were defined as the nonrapid decliner group. Patients who were classified in the rapid decliner group during follow-up period got into the end point of long-term renal prognosis study. The multivariate analysis demonstrated that AKI and preexisting CKD were risk factors for long-term renal outcomes in DKA patients (Table 3). The percentages of DKA patients in the nonrapid decliner group were stratified by non-AKI and AKI stages, as shown in Fig. 1. The long-term renal function of AKI patients deteriorated more quickly in 30 months’ time accompanying with the more advanced AKI stage.

**Table 3:** Predictors of the long-term renal outcomes in DKA patients using Cox proportional hazards model^a^

Parameters	Univariate analysis		Multivariate analysis (forward)	
	HR (95%CI)	P	HR (95%CI)	P
Male	0.868 (0.540–1.397)	0.561		
Age (year)	1.014 (1.001–1.026)	0.029		
History of CVD	1.644 (0.857–3.151)	0.134		
Type 2 diabetic mellitus	1.547 (0.940–2.545)	0.086		
Preexisting CKD	2.831 (1.647–4.868)	< 0.001	1.899 (1.088–3.314)	0.024
HbA1c(%)	1.081 (0.977–1.198)	0.132		
Baseline eGFR (ml/min/1.73m^2^)	0.990 (0.980–0.999)	0.032		
History of AKI				
Non-AKI (Reference)	Reference		Reference	
AKI	4.107 (2.273–7.421)	< 0.001	3.598 (1.957–6.614)	< 0.001

^a^-2Loglikelihood = 555.190, chi-square = 33.015; *P* < 0.001. Values express as hazard ratio (HR) and 95% confidence interval(95%CI)

### The risk factors for long-term mortality in DKA patients

**Fig. 2:**
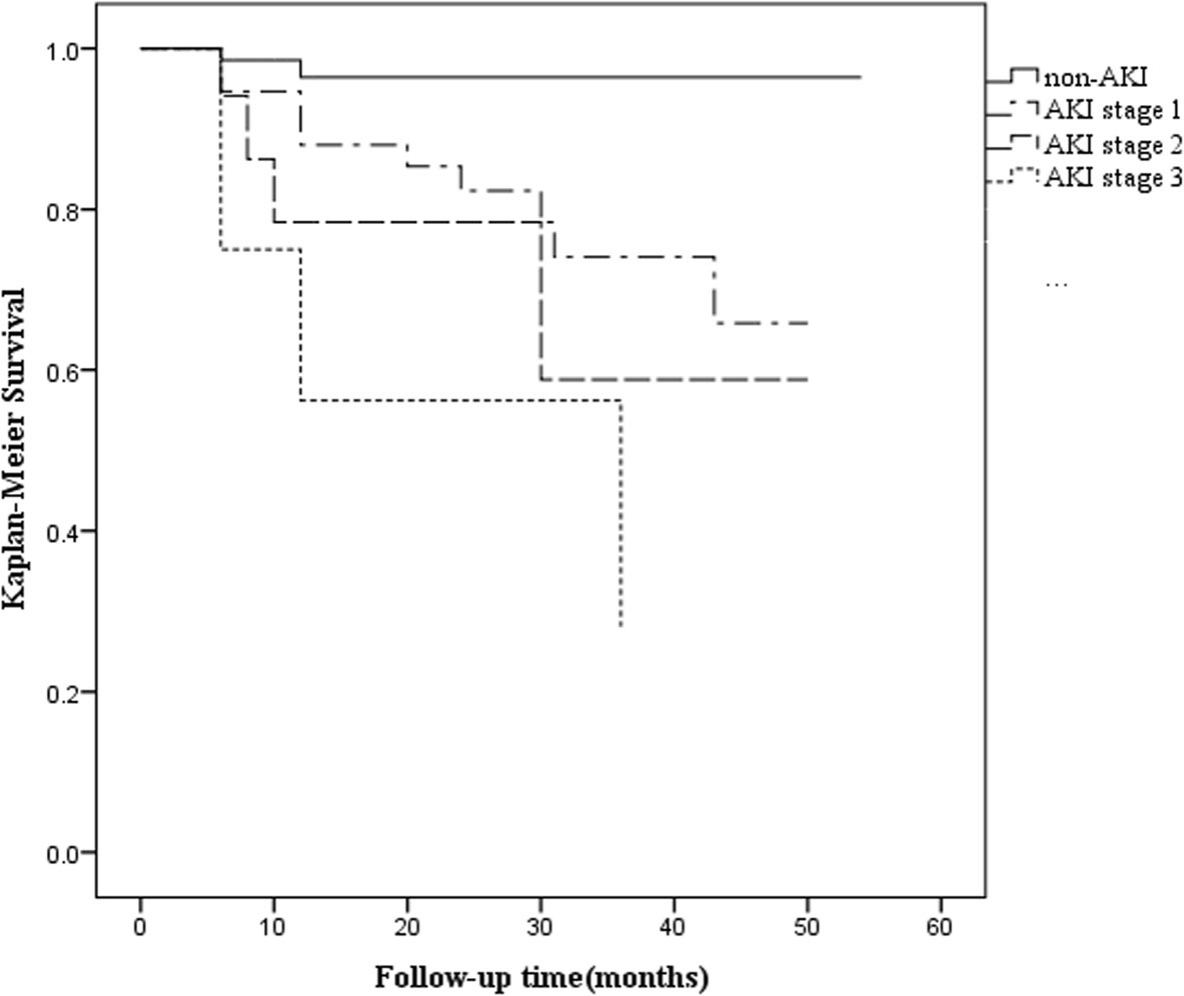
Kaplan-Meier survival analysis of DKA patients according to non-AKI and AKI stages. (Log-rank: *P* < 0.001)

During the follow-up period, a total of 21 patients died. Two of 69 (2.9%) non-AKI patients died and 19 of 82 (23.2%) AKI patients died, including 11 with stage 1 AKI (19.3% of stage 1 AKI), 4 with stage 2 AKI (23.5% of stage 2 AKI) and 4 with stage 3 AKI (50.0% of stage 3 AKI). Cox proportional hazards modeling demonstrated that age (*P* = 0.001) and AKI (*P* = 0.036) were significantly associated with long-term mortality in DKA patients (Table 4). Figure 2 showed the Kaplan-Meier survival curves of 151 DKA patients categorized into AKI and non-AKI groups. The AKI group had significantly lower survival rate than the non-AKI group. Our results also found that severe AKI stages were associated with increased long-term mortality in DKA patients.

**Table 4:** Predictors of the mortality in DKA patients using Cox proportional hazards model^a^

Parameters	Univariate analysis		Multivariate analysis (forward)	
	HR (95%CI)	P	HR (95%CI)	P
Male	1.168 (0.492–2.776)	0.725		
Age (year)	1.060 (1.031–1.090)	< 0.001	1.048 (1.020–1.078)	0.001
History of CVD	5.556 (2.328–13.262)	< 0.001		
Type 2 diabetic mellitus	3.261 (1.094–9.722)	0.034		
Preexisting CKD	1.118 (0.329–3.797)	0.858		
HbA1c (%)	0.992 (0.830–1.185)	0.927		
Baseline eGFR (ml/min/1.73m^2^)	0.972 (0.955–0.989)	0.001		
History of AKI				
Non-AKI (Reference)	Reference		Reference	
AKI	8.405 (1.956–36.121)	0.004	4.959 (1.115–22.062)	0.036

^a^-2 Log likelihood = 161.053, chi-square = 26.765; *P* < 0.001. Values express as hazard ratio (HR) and 95% confidence interval(95%CI)

## Discussion

The incidence of DM has steadily increased worldwide, and DM is becoming the leading cause of chronic noncommunicable diseases as well as the predominant pathogenic factor of CKD worldwide [11]. DKA, a severe complication of DM, leads to increased morbidity and mortality and needs to be treated immediately [3]. Recently, published data have demonstrated that AKI is a common complication of DKA and is associated with poor short-term outcomes in DKA patients [1, 3]. In this retrospective study, we documented that more than half of the DKA patients developed AKI according to the 2012 KDIGO guidelines. Increased severe renal impairment and mortality occurred in the AKI group after discharge during the long-term follow-up period. Notably, the deterioration of renal function in AKI patients was prominent between 6 months and 1 year after discharge. As mentioned previously, this is the first study to explore the influence of AKI on the long-term outcomes of DKA patients after discharge.

In clinical practice, the diagnosis and classification of AKI remains challenging, as baseline outpatient SCr before hospital admission is often unavailable. To resolve this challenge, an alternative methodology for estimating baseline Scr for AKI diagnosis and classification is proposed: estimating the baseline SCr using the Modification of Diet in Renal Disease (MDRD) formula [12], the SCr at the first documented admission (SCradm) [13] or SCrmin [14, 15] during hospitalization. These methods for estimating baseline SCr have limitations. Available evidence has shown that estimating the baseline SCr using the MDRD formula can lead to the misclassification of AKI, particularly in the early stages of AKI, and is not specific enough for an AKI diagnosis [13, 16]. A study by Edward D. Siew et al. [17] also suggested that imputed SCr based on an assumed baseline eGFR of 75 ml/min/1.73m^2^, which is recommended by the Acute Dialysis Quality Initiative (ADQI) as baseline, is improper for populations with diabetes because this method would yield low specificity for AKI and an increased false 60-day mortality rate, whereas SCradm [13] and SCrmin [14] could provide better predictive ability for long-term mortality. However, SCradm used for baseline renal function could lead to a significantly lower AKI incidence estimate than SCrmin [17]. In our study, we found that the SCr levels of inpatients gradually decreased compared to SCradm and usually decreased to the lowest level prior to discharge in most patients. Taking the abovementioned factors into consideration, we chose SCrmin in the hospital as the baseline level. Our results showed that 98 patients (54.8%) developed AKI including 66 (67%) with stage 1, 22 (22%) with stage 2, and 10 (10%) with stage 3, which is similar to the incidence rate of AKI in severe DKA patients reported by Jean-Christophe Orban et al. [3]. In this study, we also found that aging; increased Glu, SUA and WBCs; decreased pH and serum Alb; coma; and preexisting CKD were important risk factors of AKI in DKA patients according to the multivariate analysis, consistent with previous studies [3, 18–22]. Unexpectedly, more than 90% of the AKI patients were underdiagnosed and had not received optimal treatment for AKI in the hospital. These results remind us that AKI diagnosis and treatment in a practical clinical setting are unsatisfactory and that cooperation between nephrologists and non-nephrologists should be strengthened.

Our results showed that all the DKA patients, especially those in the AKI group, presented with more severe deterioration of renal function in reference to the parameters proposed by Giacomo Zoppini et al. [10]. The study by Giacomo Zoppini et al. [10] showed that the annual eGFR decline in the whole cohort was − 0.9 ± 2.9 ml/min/1.73m^2^ per year in type 2 diabetes patients; in which the average eGFR decline was − 5.8 ± 3.0 ml/min/1.73m^2^ per year and − 0.6 ± 2.0 ml/min/1.73m^2^ per year in the rapid decliners and nonrapid decliners, respectively. Our results showed that the average eGFR declined faster in the DKA patients in the AKI group than in those in the non-AKI group, at − 6.4 ± 5.0 ml/min/1.73m^2^ per year versus − 1.9 ± 3.8 ml/min/1.73m^2^ per year, respectively (*P* < 0.01), during the averaged 22 months follow-up period. Importantly, the AKI group presented a significantly rapid deterioration of eGFR, which was reduced by 10.5 ml/min/1.73m^2^, in the first 6 months to 1 year, whereas the eGFR decline rate in the non-AKI group was 4.11 ml/min/1.73m^2^ in our study (*P =* 0.001). The deterioration of renal function slowed after 1 year but remained significantly different between the AKI and non-AKI groups. Our results also demonstrated that severe AKI stages were associated with a rapid increase in renal function deterioration. Many studies have confirmed that AKI is an important risk factor for CKD. The progressive CKD prevalence is significantly higher in AKI patients than in non-AKI patients. Even with complete recovery of renal function at discharge, AKI is still a key risk factor for progressive CKD. The more advanced the AKI stage is, the higher the risk of progressive CKD becomes [23]. Possible mechanisms driving AKI transition to CKD include the following: oxidative stress, mitochondrial dysfunction, persistent chronic inflammation, endothelial dysfunction and microvascular rarefaction, incomplete regeneration of tubular cells, cell cycle arrest, DNA damage response and so on [24, 25]. Many studies have demonstrated that diabetes is an independent risk factor for AKI [26] and diabetic patients with AKI have significantly increased risk of developing into CKD due to the impaired recovery [27]. Available evidence shows that diabetes is associated with reactive oxygen species (ROS) overproduction, mitochondrial dysfunction, inflammation and hypoxia [26]. Hyperglycemia can lead to endothelial cells injury including dysfunction and apoptosis [28], and a reduction of peritubular capillaries which is associated with the decreased expression of VEGF-A [29]. Mitochondrial dysfunction has been observed in both high glucose-treated podocyte [30] and experimental or clinical diabetic kidney disease [31]. At the same time, the tubular system of DM patients, which in pathophysiology status, set the stage for development of inflammation, hypoxia and apoptosis [32]. Importantly, in vivo and in vitro studies also found that hyperglycemia can stimulate proximal tubules cells to secrete extracellular matrix via the TGF-β-dependent pathway which is the key mechanism of AKI to CKD transition [33, 34]. Moreover, it has also been observed that DKA is associated with the elevation of proinflammatory cytokines, oxidative stress [35] and elevated levels of ketones can increase the expression of adhesion molecules in endothelial cells and cause monocytes to adhere, resulting in tissue damage [36, 37]. The mechanisms mentioned above may be the possible reasons why AKI affects the long-term renal function of DKA.

In addition to the negative effects of AKI contributing to CKD, substantial evidence has demonstrated that AKI is closely associated with increased mortality, which might be partly attributed to permanent injury inflicted on other vital organs by AKI. A study showed that AKI is a significant risk factor for 2-year mortality even after complete recovery at discharge [23]. A systematic review and meta-analysis illustrated that the incidence of mortality was 8.9 per 100 person-years in survivors of AKI and 4.3 per 100 patient-years in survivors without AKI (risk ratio-RR 2.59, 95% CI 1.97–3.42) [5]. Consistent with the aforementioned studies, our results revealed that AKI was a risk factor for long-term mortality in DKA patients, as evidenced by the mortality rate, which was 8 times higher in the AKI group than in the non-AKI group; advanced AKI stages were also associated with increased mortality in DKA patients.

Since this was a single-center retrospective study, limitations to our study are inevitable. First, the number of hospitalized DKA patients included in the analysis was small. Although we collected and analyzed all DKA patients’ data in accordance with the inclusion and exclusion standards from January 2012 to January 2018, the incidence of hospitalization due to DKA linearly decreased, which could be attributed to an increase in aggressive diabetic care programs [38]. Second, most DKA patients were treated in the endocrinology and emergency departments, and detailed records of urine output were missing; therefore, we did not use urine output criteria for diagnosing AKI in this study. Inaccurate or missing urine output data reflects the real situation in non-intensive care unit (ICU) wards in the majority of hospitals in China, in accordance with the reports by Edward D. Siew et al. [17]. Some studies also pointed out that urine output might not add additional diagnostic value for mortality and worsening renal function compared with SCr in AKI patients [39]. Moreover, the Glasgow coma scale was not included in DKA patients’ history records, so we only provided only a qualitative diagnosis according to the definition of coma [6]. Third, the follow-up period was an average of 22 months for long-term outcome analyses and prognostic stratification, although we found that rapid deterioration of renal function in DKA patients with AKI mostly occurred from 6 months to 1 year after discharge and slowed thereafter. In the future, we will continue to follow these DKA patients to monitor their renal function and survival rate.

## Conclusions

AKI is a severe complication of DKA, and age; Glu, SUA and WBCs levels; pH and serum Alb; coma; and preexisting CKD are associated with AKI. AKI and severe AKI stages are associated with rapid progressive CKD and long-term mortality in DKA patients. Early recognition and prevention of AKI in hospitals and regular follow-up for the protection of renal function in DKA patients with AKI are of vital importance.

## Supplementary information

**Additional file 1: Table S1.** The changes of SCr and eGFR level between AKI and non-AKI group in DKA patients during follow-up period.

## Data Availability

The datasets used and/or analyzed during the current study available from the corresponding author on reasonable request.

## Abbreviations

AKI: Acute kidney injury
Alb: Albumin
ARF: Acute renal failure
BMI: Body mass index
CKD: Chronic kidney disease
CVD: Cardiovascular disease
DBP: Diastolic blood pressure
DKA: Diabetic ketoacidosis
DM: Diabetes mellitus
eGFR: Estimated glomerular filtration rate
Glu: Blood glucose
HbA1c: Glycosylated hemoglobin
ICU: Intensive care unit
KDIGO: Kidney disease improving global outcomes
MDRD: Modification of diet in renal disease
SBP: Systolic blood pressure
SCr: Serum creatine
Scradm: Admission SCr
Scrmin: Minimum value of SCr
SUA: Serum uric acid
WBCs: White blood cells

## References

[1] Hursh BE, Ronsley R, Islam N, Mammen C, Panagiotopoulos C (2017). Acute kidney injury in children with type 1 diabetes hospitalized for diabetic ketoacidosis. JAMA Pediatr.

[2] Realsen J, Goettle H, Chase HP (2012). Morbidity and mortality of diabetic ketoacidosis with and without insulin pump care. Diabetes Technol Ther.

[3] Orban JC, Maiziere EM, Ghaddab A, Van Obberghen E, Ichai C (2014). Incidence and characteristics of acute kidney injury in severe diabetic ketoacidosis. PLoS One.

[4] Hsu CY (2012). Yes, AKI truly leads to CKD. J Am Soc Nephrol.

[5] Coca SG, Yusuf B, Shlipak MG, Garg AX, Parikh CR (2009). Long-term risk of mortality and other adverse outcomes after acute kidney injury: a systematic review and meta-analysis. Am J Kidney Dis.

[6] Rosenberg RN (2009). Consciousness, coma, and brain death--2009. JAMA..

[7] Flamant M, Haymann JP, Vidal-Petiot E, Letavernier E, Clerici C, Boffa JJ, Vrtovsnik F (2012). GFR estimation using the Cockcroft-Gault, MDRD study, and CKD-EPI equationsin the elderly. Am J Kidney Dis.

[8] Kellum JA, Lameire N (2013). Diagnosis, evaluation, and management of acute kidney injury: a KDIGO summary (part 1). Crit Care.

[9] Kim CS, Bae EH, Ma SK, Kweon SS, Kim SW (2014). Impact of partial nephrectomy on kidney function in patients with renal cell carcinoma. BMC Nephrol.

[10] Zoppini G, Targher G, Chonchol M, Ortalda V, Negri C, Stoico V, Bonora E (2012). Predictors of estimated GFR decline in patients with type 2 diabetes and preserved kidney function. Clin J Am Soc Nephrol.

[11] Zhang L, Long J, Jiang W, Shi Y, He X, Zhou Z, Li Y, Yeung RO, Wang J, Matsushita K (2016). Trends in chronic kidney disease in China. N Engl J Med.

[12] Bellomo R, Ronco C, Kellum JA, Mehta RL, Palevsky P (2004). Acute renal failure- definition, outcome measures, animal models, fluid therapy and information technology needs: the second international consensus conference of the acute Dialysis quality initiative (ADQI) group. Crit Care.

[13] Thongprayoon C, Cheungpasitporn W, Harrison AM, Kittanamongkolchai W, Ungprasert P, Srivali N, Akhoundi A, Kashani KB (2016). The comparison of the commonly used surrogates for baseline renal function in acute kidney injury diagnosis and staging. BMC Nephrol.

[14] Thongprayoon C, Cheungpasitporn W, Kittanamongkolchai W, Srivali N, Ungprasert P, Kashani K (2015). Optimum methodology for estimating baseline serum creatinine for the acute kidney injury classification. Nephrology (Carlton).

[15] Broce JC, Price LL, Liangos O, Uhlig K, Jaber BL (2011). Hospital-acquired acute kidney injury: an analysis of nadir-to-peak serum creatinine increments stratified by baseline estimated GFR. Clin J Am Soc Nephrol.

[16] Pickering JW, Endre ZH (2010). Back-calculating baseline creatinine with MDRD misclassifies acute kidney injury in the intensive care unit. Clin J Am Soc Nephrol.

[17] Siew ED, Matheny ME, Ikizler TA, Lewis JB, Miller RA, Waitman LR, Go AS, Parikh CR, Peterson JF (2010). Commonly used surrogates for baseline renal function affect the classification and prognosis of acute kidney injury. Kidney Int.

[18] Chawla LS, Eggers PW, Star RA, Kimmel PL (2014). Acute kidney injury and chronic kidney disease as interconnected syndromes. N Engl J Med.

[19] Barski L, Nevzorov R, Rabaev E, Jotkowitz A, Harman-Boehm I, Zektser M, Zeller L, Shleyfer E, Almog Y (2012). Diabetic ketoacidosis: clinical characteristics, precipitating factors and outcomes of care. Isr Med Assoc J.

[20] Bagshaw SM, George C, Bellomo R (2008). Early acute kidney injury and sepsis: a multicentre evaluation. Crit Care.

[21] Park SH, Shin WY, Lee EY, Gil HW, Lee SW, Lee SJ, Jin DK, Hong SY (2011). The impact of hyperuricemia on in-hospital mortality and incidence of acute kidney injury in patients undergoing percutaneous coronary intervention. Circ J.

[22] Wiedermann CJ, Wiedermann W, Joannidis M (2010). Hypoalbuminemia and acute kidney injury: a meta-analysis of observational clinical studies. Intensive Care Med.

[23] Xu JR, Zhu JM, Jiang J, Ding XQ, Fang Y, Shen B, Liu ZH, Zou JZ, Liu L, Wang CS (2015). Risk factors for Long-term mortality and progressive chronic kidney disease associated with acute kidney injury after cardiac surgery. Medicine (Baltimore).

[24] Fiorentino M, Grandaliano G, Gesualdo L, Castellano G (2018). Acute kidney injury to chronic kidney disease transition. Contrib Nephrol.

[25] Basile DP, Bonventre JV, Mehta R, Nangaku M, Unwin R, Rosner MH, Kellum JA, Ronco C (2016). ADQI XIII work group: progression after AKI: understanding maladaptive repair processes to predict and identify therapeutic treatments. J Am Soc Nephrol.

[26] Yu SMW, Bonventre JV (2018). Acute kidney injury and progression of diabetic kidney disease. Adv Chronic Kidney Dis.

[27] Thakar CV, Christianson A, Himmelfarb J, Leonard AC (2011). Acute kidney injury episodes and chronic kidney disease risk in diabetes mellitus. Clin J Am Soc Nephrol.

[28] Ho FM, Lin WW, Chen BC, Chao CM, Yang CR, Lin LY, Lai CC, Liu SH, Liau CS (2006). High glucose-induced apoptosis in human vascular endothelial cells is mediated through NF-κB and c-Jun NH_2_-terminal kinase pathway and prevented by PI3K/Akt/eNOS pathway. Cell Signal.

[29] Lindenmeyer MT, Kretzler M, Boucherot A, Berra S, Yasuda Y, Henger A, Eichinger F, Gaiser S, Schmid H, Rastaldi MP (2007). Interstitial vascular rarefaction and reduced VEGF-A expression in human diabetic nephropathy. J Am Soc Nephrol.

[30] Feng J, Ma YQ, Chen ZW, Hu JJ, Yang Q (2019). Ding GH:mitochondrial pyruvate carrier 2 mediates mitochondrial dysfunction and apoptosis in high glucose-treated podocytes. Life Sci.

[31] Ducasa GM, Mitrofanova A (2019). FornoniA:crosstalk between lipids and mitochondria in diabetic kidney disease. Curr Diab Rep.

[32] VallonV TSC (2012). Renal function in diabetic disease models: the tubular system in the pathophysiology of the diabetic kidney. Annu Rev Physiol.

[33] Rocco MV, Chen Y, Goldfarb S, Ziyadeh FN (1992). Elevated glucose stimulates TGF-beta gene expression and bioactivity in proximal tubule. Kidney Int.

[34] Chang AS, Hathaway CK, Smithies O, Kakoki M (2016). Transforming growth factor-beta1 and diabetic nephropathy. Am J Physiol Renal Physiol.

[35] Stentz FB, Umpierrez GE, Cuervo R, Kitabchi AE (2004). Proinflammatory cytokines, markers of cardiovascular risks, oxidative stress, and lipid peroxidation in patients with hyperglycemic crises. Diabetes..

[36] Kanikarla-Marie P, Jain SK (2016). Hyperketonemia and ketosis increase the risk of complications in type 1 diabetes. Free Radic Biol Med.

[37] Kanikarla-Marie P, Jain SK (2015). Hyperketonemia (acetoacetate) upregulates NADPH oxidase 4 and elevates oxidative stress, ICAM-1, and monocyte adhesivity in endothelial cells. Cell Physiol Biochem.

[38] Liu CC, Chen KR, Chen HF, Huang HL, Ko MC, Li CY (2010). Trends in hospitalization for diabetic ketoacidosis in diabetic patients in Taiwan: analysis of national claims data, 1997-2005. J Formos Med Assoc.

[39] Lopes JA, Fernandes P, Jorge S, Goncalves S, Alvarez A, Costa ESZ, Franca C, Prata MM (2008). Acute kidney injury in intensive care unit patients: a comparison between the RIFLE and the acute kidney injury network classifications. Crit Care.

