# Supplementary material for: The incidence, risk factors, and long-term outcomes of acute kidney injury in hospitalized diabetic ketoacidosis patients
Source: BMC Nephrol. 2020 Feb 12;21:48. doi: 10.1186/s12882-020-1709-z (PMC7017527; doi:10.1186/s12882-020-1709-z)
Supplement: Supplementary file 1 — Additional file 1: Table S1. The changes of SCr and eGFR level between AKI and non-AKI group in DKA patients during follow-up period. [file 12882_2020_1709_MOESM1_ESM.doc]

Table S1. The changes of SCr and eGFR level which based on self baseline SCr and eGFR at discharge between AKI and non-AKI group in diabetic ketoacidosis patients during follow-up period

|  | SCr  (umol/L) | |  | eGFR  (ml/min/1.73m^2^) | |  |
| --- | --- | --- | --- | --- | --- | --- |
| Follow-up time | AKI group | Non-AKI group | P | AKI group | Non-AKI group | P |
| 6 month-1 year | 13.5  [5.75-34.25]  (n=22) | 4.5  [2.0-8.5]  (n=24) | 0.001 | -10.5  [-13.64- -6.52]  (n=22) | -4.11  [-8.0- -0.25]  (n=24) | 0.001 |
| 1 year- 2 year | 12.5  [7.0-48.75]  (n=28) | 5.0  [-2.75-8.00]  (n=28) | 0.003 | -12.15  [-17.56- -8.25]  (n=28) | -4.65  [-6.84-1.75]  (n=28) | <0.001 |
| 2 year-3 year | 20.0  [11.0-47.0]  (n=25) | 5.5  [2.00-10.00] (n=22) | 0.002 | -13.32  [-21.70- -8.64]  (n=25) | -4.90  [-8.45- 3.38] (n=22) | <0.001 |
| 3 year | 23.0  [12.75-52.00]  (n=18) | 6.50  [1.00-13.00]  (n=14) | 0.016 | -13.66  [-20.32- -10.89]  (n=18) | -5.26  [-13.97--2.32]  (n=14) | 0.002 |
